# Supplementary figures and images for: Backbone: An R package for extracting the backbone of bipartite projections
Source: PLoS One. 2021 Jan 6;16(1):e0244363. doi: 10.1371/journal.pone.0244363 (PMC7787471; doi:10.1371/journal.pone.0244363)

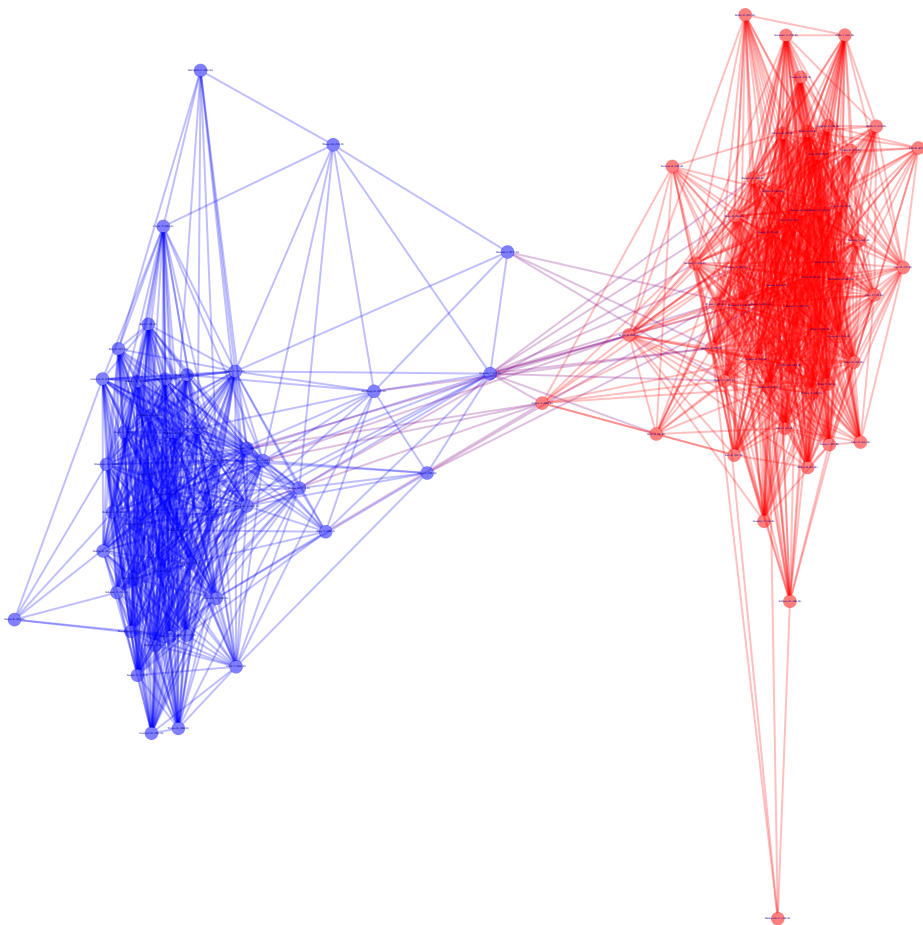

Supplement: S1 File — (ZIP) [file pone.0244363.s001.zip › figures/fdsm_bb.pdf]

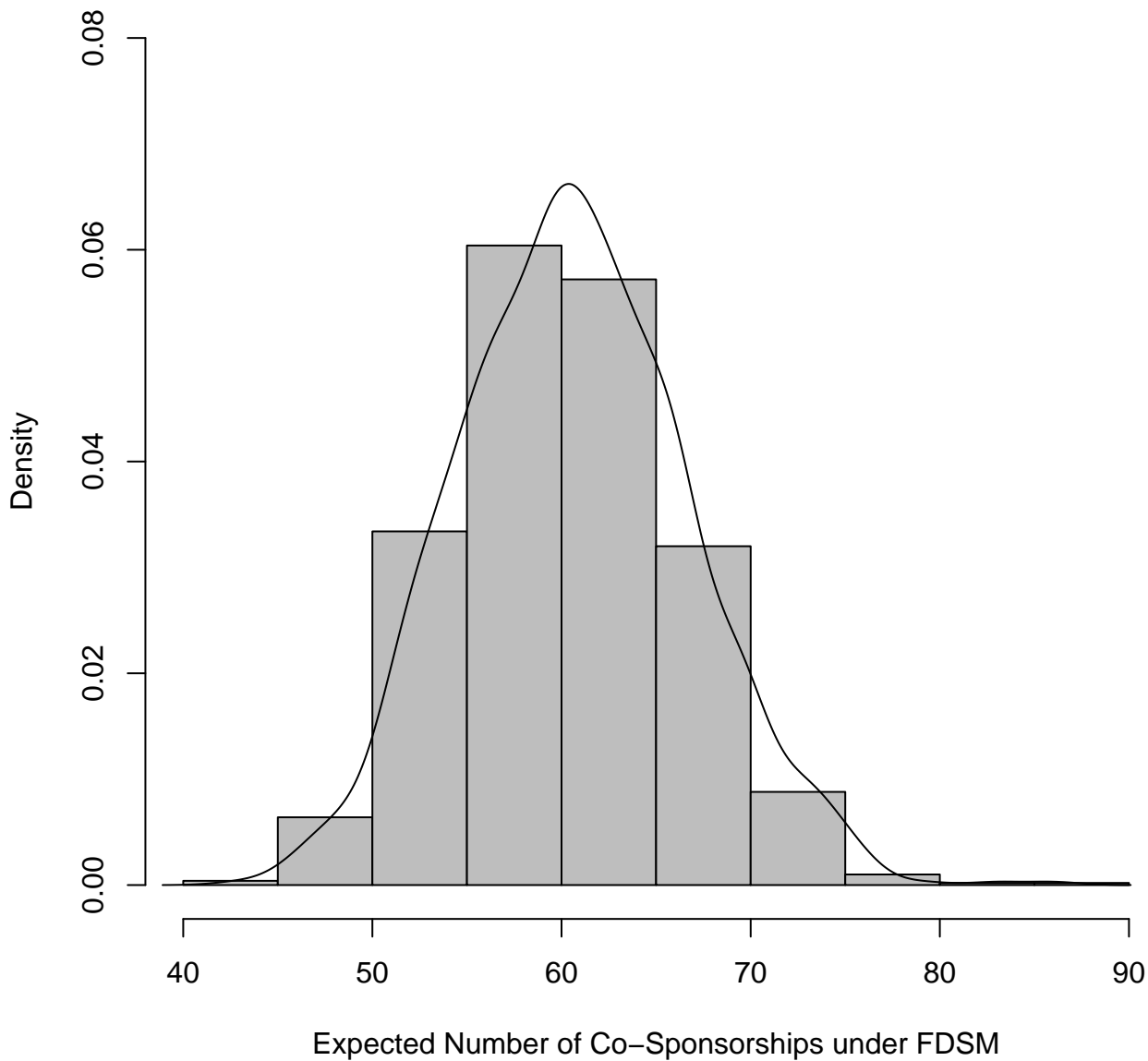

Supplement: S1 File — (ZIP) [file pone.0244363.s001.zip › figures/fdsm_histogram.pdf]

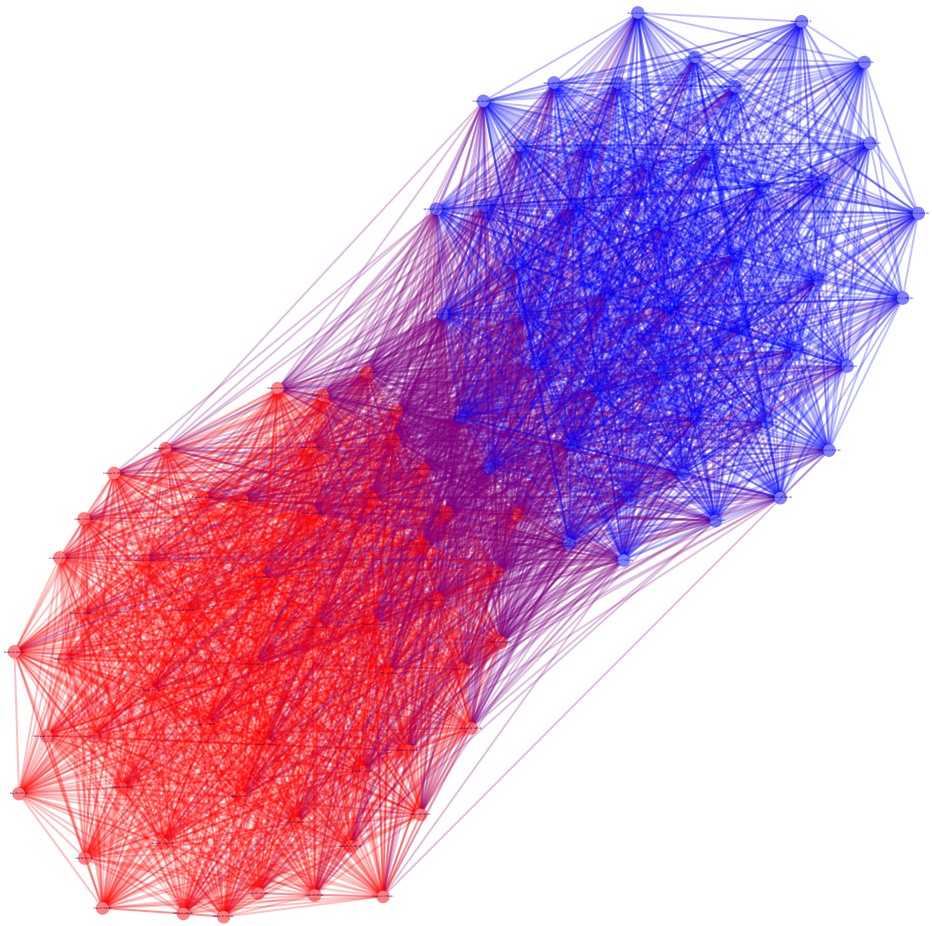

Supplement: S1 File — (ZIP) [file pone.0244363.s001.zip › figures/hyperg_bb.pdf]

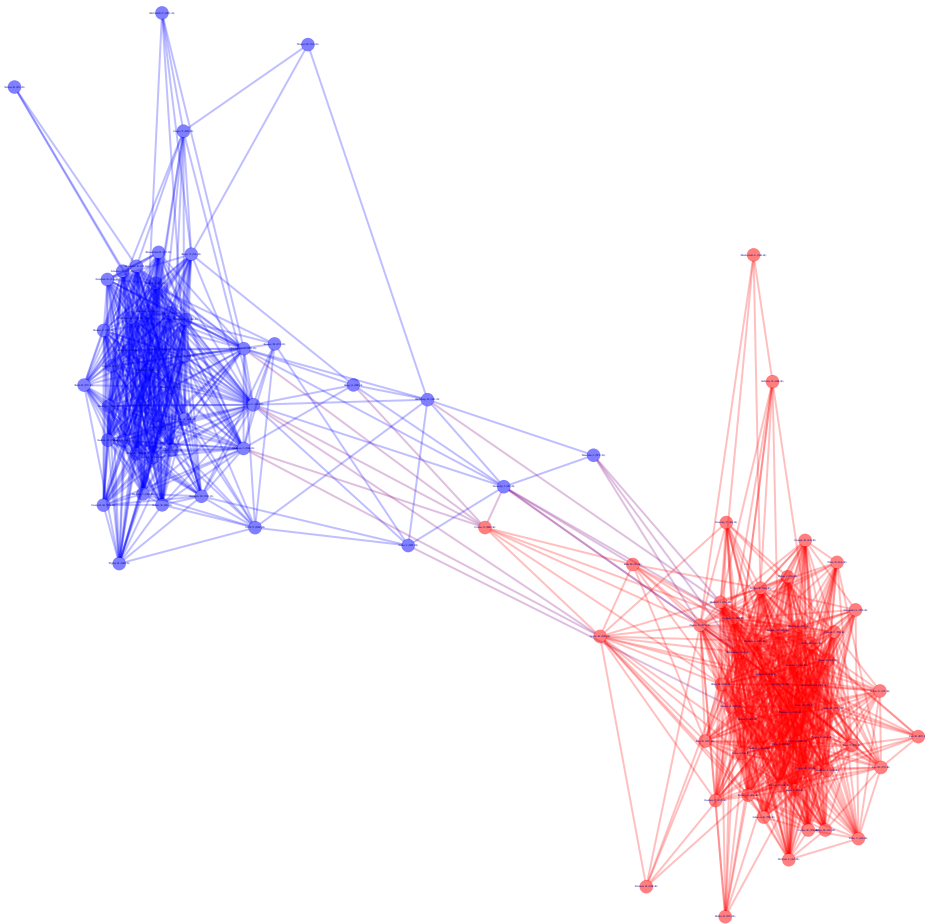

Supplement: S1 File — (ZIP) [file pone.0244363.s001.zip › figures/sdsm_bb.pdf]

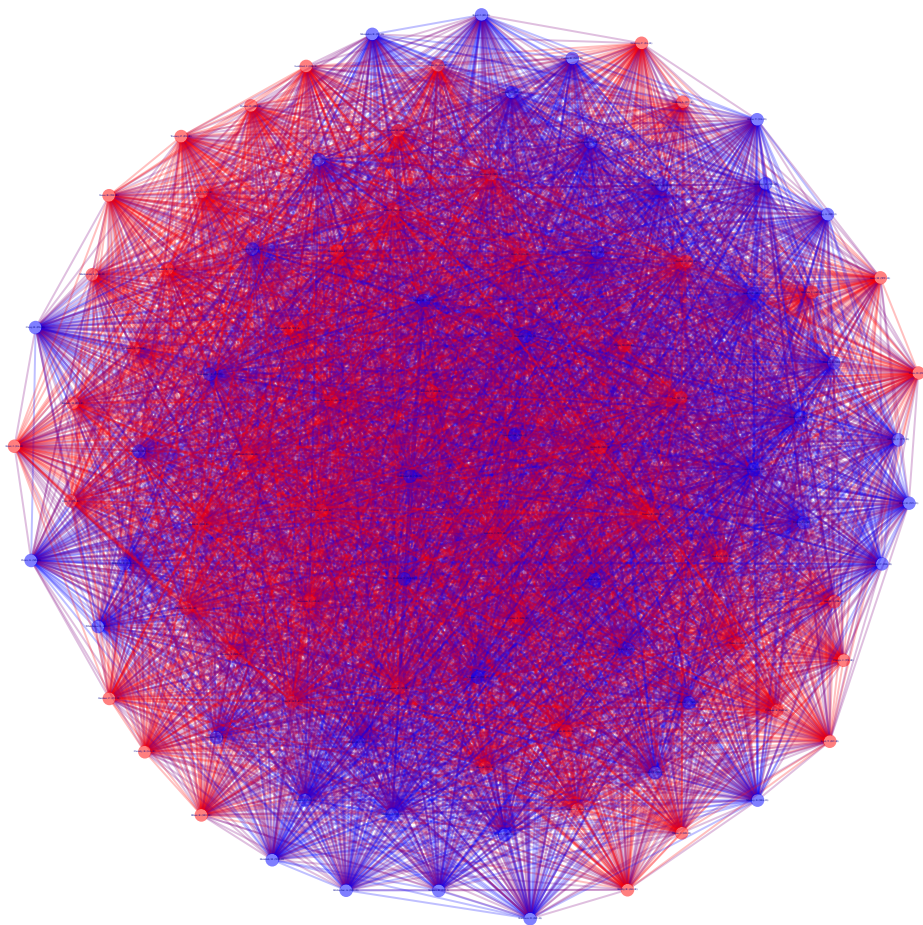

Supplement: S1 File — (ZIP) [file pone.0244363.s001.zip › figures/universal_bb1.pdf]

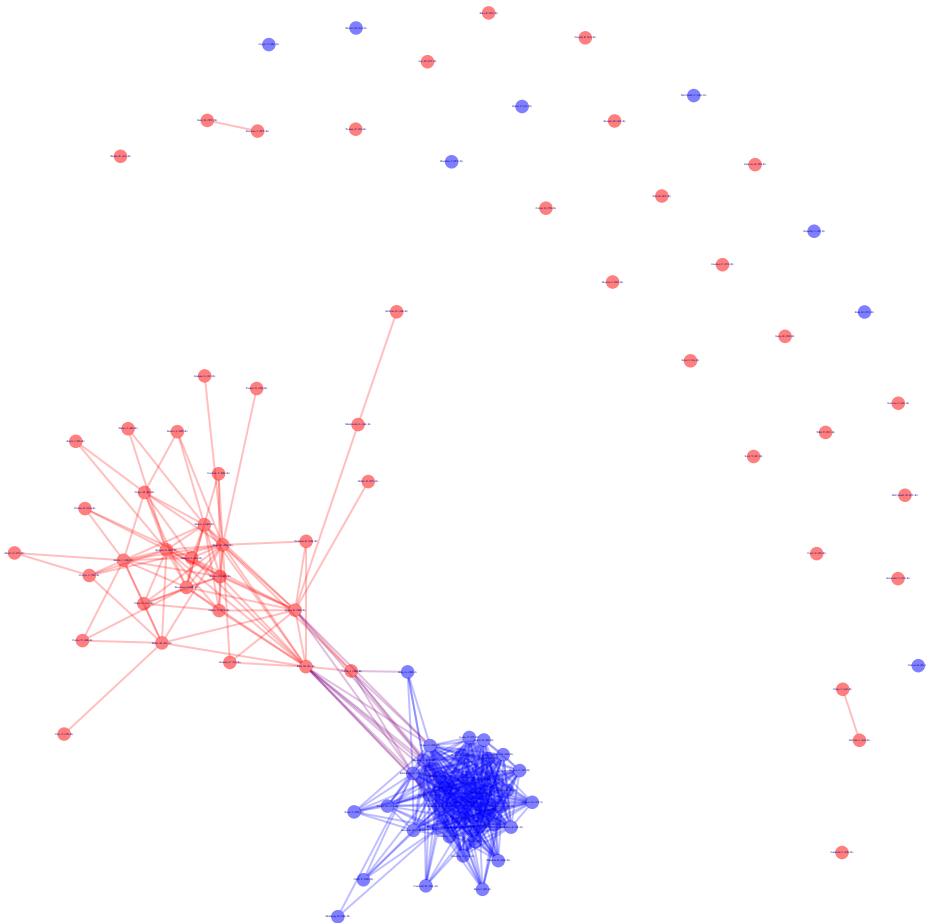

Supplement: S1 File — (ZIP) [file pone.0244363.s001.zip › figures/universal_bb2.pdf]
