# Supplementary material for: Backbone: An R package for extracting the backbone of bipartite projections
Source: PLoS One. 2021 Jan 6;16(1):e0244363. doi: 10.1371/journal.pone.0244363 (PMC7787471; doi:10.1371/journal.pone.0244363)
Supplement: Attachment — Submitted filename: PONE-D-20-23636_reviewer.pdf [file pone.0244363.s002.pdf]

PLOS ONE

backbone: An R package for extracting the backbone of bipartite projections

--Manuscript Draft--

**Manuscript Number:**	PONE-D-20-23636
**Article Type:**	Research Article
**Full Title:**	backbone: An R package for extracting the backbone of bipartite projections
**Short Title:**	backbone: An R package for extracting the backbone of bipartite projections
**Corresponding Author:**	Zachary P. Neal<br>Michigan State University<br>East Lansing, MI UNITED STATES
**Keywords:**	network; bipartite projection
**Abstract:**	Bipartite projections are used in a wide range of network contexts including politics (bill co-sponsorship), genetics (gene co-expression), economics (executive board co-membership), and innovation (patent co-authorship). However, because bipartite projections are always weighted graphs, which are inherently challenging to analyze and visualize, it is often useful to examine the `backbone,' an unweighted subgraph containing only the most significant edges. In this paper, we introduce the R package backbone for extracting the backbone of weighted bipartite projections, and use bill sponsorship data from the 114th session of the United States Senate to demonstrate its functionality.
**Order of Authors:**	Rachel Domagalski
	Zachary P. Neal
	Bruce Sagan
**Additional Information:**	
**Question**	**Response**
**Financial Disclosure**<br><br>Enter a financial disclosure statement that describes the sources of funding for the work included in this submission. Review the submission guidelines for detailed requirements. View published research articles from *PLOS ONE* for specific examples.<br><br>This statement is required for submission and **will appear in the published article** if the submission is accepted. Please make sure it is accurate.	ZN and BS received funding from the National Science Foundation (#1851625 & #2016320).<br><br>The funders had no role in study design, data collection and analysis, decision to publish, or preparation of the manuscript.

Powered by Editorial Manager® and ProduXion Manager® from Aries Systems Corporation

**Unfunded studies**<br>Enter: *The author(s) received no specific funding for this work.*<br><br>**Funded studies**<br>Enter a statement with the following details:<br>• Initials of the authors who received each award<br>• Grant numbers awarded to each author<br>• The full name of each funder<br>• URL of each funder website<br>• Did the sponsors or funders play any role in the study design, data collection and analysis, decision to publish, or preparation of the manuscript?<br>• **NO** - Include this sentence at the end of your statement: *The funders had no role in study design, data collection and analysis, decision to publish, or preparation of the manuscript.*<br>• **YES** - Specify the role(s) played.<br><br>* typeset	
**Competing Interests**<br><br>Use the instructions below to enter a competing interest statement for this submission. On behalf of all authors, disclose any competing interests that could be perceived to bias this work—acknowledging all financial support and any other relevant financial or non-financial competing interests.<br><br>This statement **will appear in the published article** if the submission is accepted. Please make sure it is accurate. View published research articles from *PLOS ONE* for specific examples.	The authors have declared that no competing interests exist.

Powered by Editorial Manager® and ProduXion Manager® from Aries Systems Corporation

**NO authors have competing interests**<br><br>Enter: *The authors have declared that no competing interests exist.*<br><br>**Authors with competing interests**<br><br>Enter competing interest details beginning with this statement:<br><br>*I have read the journal's policy and the authors of this manuscript have the following competing interests: [insert competing interests here]*<br><br>* typeset	
**Ethics Statement**<br><br>Enter an ethics statement for this submission. This statement is required if the study involved:<br><br>• Human participants<br>• Human specimens or tissue<br>• Vertebrate animals or cephalopods<br>• Vertebrate embryos or tissues<br>• Field research<br><br>Write "N/A" if the submission does not require an ethics statement.<br><br>General guidance is provided below. Consult the submission guidelines for detailed instructions. **Make sure that all information entered here is included in the Methods section of the manuscript.**	N/A

Powered by Editorial Manager® and ProduXion Manager® from Aries Systems Corporation

**Format for specific study types**<br><br>**Human Subject Research (involving human participants and/or tissue)**<br>• Give the name of the institutional review board or ethics committee that approved the study<br>• Include the approval number and/or a statement indicating approval of this research<br>• Indicate the form of consent obtained (written/oral) or the reason that consent was not obtained (e.g. the data were analyzed anonymously)<br><br>**Animal Research (involving vertebrate animals, embryos or tissues)**<br>• Provide the name of the Institutional Animal Care and Use Committee (IACUC) or other relevant ethics board that reviewed the study protocol, and indicate whether they approved this research or granted a formal waiver of ethical approval<br>• Include an approval number if one was obtained<br>• If the study involved *non-human primates*, add *additional details* about animal welfare and steps taken to ameliorate suffering<br>• If anesthesia, euthanasia, or any kind of animal sacrifice is part of the study, include briefly which substances and/or methods were applied<br><br>**Field Research**<br><br>Include the following details if this study involves the collection of plant, animal, or other materials from a natural setting:<br>• Field permit number<br>• Name of the institution or relevant body that granted permission	
**Data Availability**<br><br>Authors are required to make all data underlying the findings described fully available, without restriction, and from the time of publication. PLOS allows rare exceptions to address legal and ethical concerns. See the PLOS Data Policy and FAQ for detailed information.	Yes - all data are fully available without restriction

A Data Availability Statement describing where the data can be found is required at submission. Your answers to this question constitute the Data Availability Statement and **will be published in the article**, if accepted.<br><br>**Important:** Stating 'data available on request from the author' is not sufficient. If your data are only available upon request, select 'No' for the first question and explain your exceptional situation in the text box.<br><br>Do the authors confirm that all data underlying the findings described in their manuscript are fully available without restriction?	
**Describe where the data may be found in full sentences. If you are copying our sample text, replace any instances of XXX with the appropriate details.**<br><br>• If the data are **held or will be held in a public repository**, include URLs, accession numbers or DOIs. If this information will only be available after acceptance, indicate this by ticking the box below. For example: *All XXX files are available from the XXX database (accession number(s) XXX, XXX.*).<br>• If the data are all contained **within the manuscript and/or Supporting Information files**, enter the following: *All relevant data are within the manuscript and its Supporting Information files.*<br>• If neither of these applies but you are able to provide **details of access elsewhere**, with or without limitations, please do so. For example:<br><br>*Data cannot be shared publicly because of [XXX]. Data are available from the XXX Institutional Data Access / Ethics Committee (contact via XXX) for researchers who meet the criteria for access to confidential data.*<br><br>*The data underlying the results presented in the study are available from (include the name of the third party*	The data and code will be held in the OSF public repository.

and contact information or URL).<br>• This text is appropriate if the data are owned by a third party and authors do not have permission to share the data.<br><br>* typeset	
Additional data availability information:	Tick here if the URLs/accession numbers/DOIs will be available only after acceptance of the manuscript for publication so that we can ensure their inclusion before publication.

Powered by Editorial Manager® and ProduXion Manager® from Aries Systems Corporation

Cover Letter

backbone: An R package for extracting the backbone of bipartite projections

Rachel Domagalski[1], Zachary P. Neal[2*], Bruce Sagan[1],

1 Department of Mathematics, Michigan State University, East Lansing, Michigan, USA
2 Department of Psychology, Michigan State University, East Lansing, Michigan, USA

* zpneal@msu.edu

Abstract

Bipartite projections are used in a wide range of network contexts including politics (bill co-sponsorship), genetics (gene co-expression), economics (executive board co-membership), and innovation (patent co-authorship). However, because bipartite projections are always weighted graphs, which are inherently challenging to analyze and visualize, it is often useful to examine the 'backbone,' an unweighted subgraph containing only the most significant edges. In this paper, we introduce the R package `backbone` for extracting the backbone of weighted bipartite projections, and use bill sponsorship data from the 114th session of the United States Senate to demonstrate its functionality.

Introduction

Networks are useful for studying many different phenomena in the natural and social worlds, but network data can be difficult to collect directly. As a result, it is common for research to measure an unobserved unipartite network of interest using a bipartite projection in which the edges capture whether (or the extent to which) two vertices co-participate in a relevant event. For example, friendship networks are measured using event co-attendance [1], political networks are measured using bill co-sponsorship [2], executive networks are measured using board co-membership [3], scholarly collaboration networks are measured using paper co-authorship [4], knowledge networks are measured using paper co-citation [5], and genetic networks are measured using gene co-expression [6].

However, there are several challenges to studying bipartite projections. First, the projection function "transforms the problem of analysing a bipartite structure into the problem of analysing a weighted one, which is not easier" [7, p. 34-35]. Second, when transforming a bipartite graph into a unipartite graph via projection, information about the 'events' responsible for edges between vertices is lost [7]. Third, bipartite projections introduce topological artifacts such as inflated clustering, such that the projection of "even a random [bipartite] network – one that has no particular structure built into it at all – will be highly clustered" [8, p. 128]. Finally, the scale of each edge weight in a bipartite projection is driven by characteristics of both sets of vertices in the original bipartite graph. For example, observing two people attending many of the same events (i.e. a large edge weight) may still be uninteresting if they each attended many large events, but observing two people attend just a few of the same events (i.e. a small edge weight) may be interesting if they each attended only a few small events [9]

For these reasons, it is often useful to extract and study the *backbone* of a bipartite projection. The backbone is an unweighted (i.e. an edge is either present or absent) or signed (i.e. an edge can be positive, negative, or absent) graph that preserves only the most “significant” edges from the weighted bipartite projection. Multiple methods of backbone extraction exist for bipartite projections [9], but until now there has not been a single software package that implements the most common methods. In this paper, we introduce and demonstrate the `backbone` R package, which implements four backbone extraction methods – a universal threshold, a hypergeometric model, a stochastic degree sequence model, and a fixed degree sequence model – in a common framework that facilitates their use. It can be installed in R [10] from The Comprehensive R Archive Network (CRAN) via `install.packages("backbone")` and used with `library(backbone)` [11]. This manuscript is based on version `backbone 1.2.0`. For further information regarding the CRAN distribution, please visit https://CRAN.R-project.org/package=backbone. For additional materials relating to `backbone`, including papers, presentations, workshop materials, and data sets, please visit http://www.zacharyneal.com/backbone.

Graph theory preliminaries

A *graph* G is a set of objects called *vertices*, together with a set of 2-element subsets of the vertices which are called *edges*. An edge between vertices i and j can be denoted as $e = ij$. If there exists an edge $e = ij$ between vertices i and j, we say that i and j are *adjacent*. The *degree* of vertex i is the number of edges of the form ij for some j. The *adjacency matrix* of a graph G with n vertices is an $n \times n$ matrix $G = [G_{ij}]$ where $G_{ij} = 1$ if an edge is present between vertex i and vertex j, or $G_{ij} = 0$ if that edge is absent. We call a graph *weighted* if each edge has an associated numeric value, and unweighted otherwise. In the weighted case $G_{ij} = w(ij)$ where $w(ij)$ is the weight of the edge $e = ij$. We make no distinction between a graph and its adjacency matrix.

We call a graph *bipartite* if the set of vertices can be partitioned into two sets U and W such that each edge of the graph is of the form ij where $i \in U$ and $j \in W$. The sets U and W are called *independent*, meaning there are no edges present within the sets. These graphs can be represented by a *biadjacency* matrix, B. This matrix uses rows to represent vertices in the set U and columns to represent vertices in the set W. We set $B_{ij} = 1$ if there is an edge between vertex i of U and vertex j of W, and set $B_{ij} = 0$ otherwise. As with graphs, we conflate a bipartite graph with its biadjacency matrix. The row sum of the ith row of the biadjacency matrix, denoted R_i, is equal to the degree of the ith vertex in the set U. Similarly, the column sum of the j column of B, denoted C_j, is the degree of the jth vertex in W.

We can use bipartite graphs to study social networks where the vertices of U are agents (e.g., people), the vertices of W are artifacts (e.g., events), and edges represent the affiliation of a person with an artifact. These bipartite networks are often also called affiliation networks or two-mode networks. To transform this data into a weighted graph, we *project* the bipartite adjacency matrix B by multiplying it by its transpose $B^\top$. This produces a weighted graph $G = BB^\top$ called the *bipartite projection*, where G_{ij} is equal to the number of artifacts of W with which both i and j are affiliated when $i \neq j$. The value G_{ii} is equal to the total number of artifacts with which i is affiliated. Let

$$M_{ij} = \min(G_{ii}, G_{jj}) - (|W| - \max(G_{ii}, G_{jj})).$$

The value of each off-diagonal entry G_{ij} is bounded by

$$\max(0, M_{ij}) \leq G_{ij} \leq \min(G_{ii}, G_{jj}).$$

Bipartite projections are of interest in social network analysis because they allow us to construct a network from artifact affiliations, which are often easier to obtain than taking a survey of the network members. If the number of members of the network is large, getting complete and reliable information regarding relationships between members can involve long and repetitive survey techniques which can lead to survey fatigue and costly field work. Additionally, in some settings, individuals may be reluctant to provide information about their relationships. In these cases, it is often beneficial and easier to collect bipartite network information, such as event attendance, then project to infer a weighted network between the social figures.

Because bipartite projections are weighted, and because what counts as a 'large' or 'small' weight can differ for each edge, it can be useful to reduce this information by focusing on an unweighted subgraph that contains only the most important edges. We call this subgraph a *backbone* of G, which we denote as G'.

Backbone extraction methods

The simplest approach to extracting a backbone of bipartite projections applies a single, universal threshold T to all edges such that:

$$G'_{ij} = \begin{cases} 1 & \text{if } G_{ij} > T \\ 0 & \text{if } G_{ij} \leq T \end{cases}.$$

For example, a threshold value of $T = 0$ indicates that as long as a pair of vertices are affiliated with at least one of the same artifacts (i.e. they have a nonzero edge weight), an edge between them will be present in the backbone. We call this type of backbone *binary*. It is also possible to extract a *signed* backbone by selecting distinct upper T^+ and lower T^- thresholds, $T^- < T^+$, such that

$$G'_{ij} = \begin{cases} 1 & \text{if } G_{ij} > T^+ \\ -1 & \text{if } G_{ij} < T^- \\ 0 & \text{if } T^- \leq G_{ij} \leq T^+ \end{cases}.$$

However, backbones extracted using universal thresholds are very dense and clustered, and therefore tend to be uninformative [7, 9].

An alternative approach to backbone extraction, and the approach that `backbone` is designed to facilitate, identifies important edges to be retained in the backbone using a statistical test that compares an edge's observed weight to the distribution of its weights under a null model. Given a null model that can generate a distribution of an edge's weights G^*_{ij}, it is possible to compute the probability that an edge's observed weight is in the upper ($P(G^*_{ij} \geq G_{ij})$) or lower ($P(G^*_{ij} \leq G_{ij})$) tail of this distribution, and therefore not likely to be the result of such a null model. Using these probabilities and a significance level α for the test, a *signed* backbone can be extracted such that:

$$G'_{ij} = \begin{cases} 1 & \text{if } P(G^*_{ij} \geq G_{ij}) < \alpha/2 \\ -1 & \text{if } P(G^*_{ij} \leq G_{ij}) < \alpha/2 \\ 0 & \text{otherwise} \end{cases}.$$

In many cases, a simpler *binary* backbone may be more useful, which can be achieved by discarding the negative edges such that:

$$G'_{ij} = \begin{cases} 1 & \text{if } P(G^*_{ij} \geq G_{ij}) < \alpha/2 \\ 0 & \text{if } P(G^*_{ij} \geq G_{ij}) \geq \alpha/2 \end{cases}.$$

However, whether a binary or signed backbone is extracted, a two-tailed significance test is used because, for any given edge, the observed value in the projection could be in either tail of the null distribution.

The challenge to using the statistical approach to backbone extraction lies in defining a suitable null model and computing the required probabilities (i.e. edge-wise p-values). A family of nine null models can be defined by the constraints they place on the row and column sums in a random bipartite graph B^*: The row sums in B^*, and separately the column sums in B^*, can be unconstrained, constrained to match those in B *on average*, or constrained to match those in B *exactly* [12, 13]. Let $\mathcal{R}$ be a set of restrictions on the row and column vertex degrees corresponding to one of these nine possibilities, and let $\mathcal{B}(\mathcal{R})$ be the space of all bipartite graphs B^* which satisfy those conditions. This approach to backbone extraction compares the values G_{ij}, the bipartite projection of interest, to the distributions that describe $G^*_{ij} = (B^* B^{*\top})_{ij}$ for all bipartite graphs $B^* \in \mathcal{B}(\mathcal{R})$. Here, we focus on three sets of restrictions $\mathcal{B}(\mathcal{R})$, defining three distinct null models: (1) the hypergeometric model, in which row sums are fixed but column sums are unconstrained; (2) the stochastic degree sequence model, in which both row and column sums are proportionally constrained; and (3) the fixed degree sequence mode, in which both row and column sums are fixed.

Hypergeometric Model (HM)

The hypergeometric model constrains the row sums in B^* as equal to their values in B (i.e. fixed), but places no constraints on the column sums [14, 15]. The distribution of $(B^* B^{*\top})_{ij}$ for all bipartite graphs $B^* \in \mathcal{B}(\mathcal{R})$ is given by the hypergeometric distribution.

The hypergeometric distribution is a discrete probability distribution that models the probability of having k "successes" in a random sample of size n (drawn without replacement) from a population of size N, where K of the objects are considered successes. The probability mass function is given by

$$Pr(X = k) = \frac{\binom{K}{k}\binom{N-K}{n-k}}{\binom{N}{n}}$$

where X is the random variable of the distribution.

In the case of a bipartite projection, let B^* be a bipartite graph with independent sets U and W. Let $U = \{u_1, u_2, \ldots, u_m\}$. We denote the neighborhood of a vertex i by $N(i)$, meaning the set of vertices to which i is adjacent. Then entry $G^*_{ij} = (B^* B^{*\top})_{ij}$ is the number of vertices in $N(u_i)$ which are also in $N(v_j)$. So the corresponding distribution is hypergeometric with population $N = |W|$, sample size $n = |N(u_i)|$, $K = |N(u_j)|$ possible "successful" objects, and $k = |N(u_i) \cap N(u_j)|$ successes.

This method ensures that both i and j are affiliated with the same number of artifacts we've observed in the original data, but the number of individuals that are affiliated with each artifact may vary. From the probability mass function we can find the probability of i and j participating in at least G_{ij} events (for positive backbone edges) or at most G_{ij} events (for negative backbone edges).

Stochastic Degree Sequence Model (SDSM)

The stochastic degree sequence model constrains B^* so that its *expected* row sums and *expected* column sums equal those observed in B [9]. That is, for any given B^*, each row and column sum may be higher or lower than its observed degree in B, but on average they are equ[illegible] When B^* is generated by filling the B^*_{ij} with the outcomes of independent Bernoulli trials, the distribution of $(B^* B^{*\top})_{ij}$ for all bipartite graphs

$B^* \in \mathcal{B}(\mathcal{R})$ is given by a Poisson binomial distribution [16][1]. A *Bernoulli trial* is a random variable with exactly two outcomes, often referred to as "success" and "failure", or "1" and "0." The Poisson binomial distribution is the distribution of a sum of independent Bernoulli trials. We let p_k be the probability of success of the kth trial and call the p_k the *parameters* of the distribution. When the probability of success on each Bernoulli trial is equal, this reduces to the ordinary binomial distribution.

The Poisson binomial distribution models the probability of getting k successes in n trials, where each trial has a specific probability for success [16, 17]. To apply the Poisson binomial distribution to compute $P(G^*_{ij} \geq G_{ij})$ we must first determine the independent probabilities $P(B^*_{ij} = 1)$. There are several ways to determine $P(B^*_{ij} = 1)$, including simple equations such as $(R_i \times C_j) \setminus \sum B$ where $\sum B$ is the sum of each entry of the biadjacency matrix of B [13, 16][2], and predicted probabilities from binary outcome models [9]. These older methods are available in the `backbone` package, but here we introduce and describe the *polytope* method [18].

The polytope method revolves around creating a convex set. A subset C of $\mathbb{R}^n$ is *convex* if for any $a, b \in C$, the line segment joining a and b is also in C. The *convex hull* of a set points $A \subseteq \mathbb{R}^n$, $conv(A)$, is the smallest convex set containing A. When A is a finite set, the convex hull of A is called the *polytope* generated by A.

Consider all $m \times n$ matrices in $\mathcal{B}(\mathcal{R})$ as vectors in $\mathbb{R}^{mn}$, where $\mathcal{B}(\mathcal{R})$ is all zero-one matrices with the same row and column sums as B. Let this set of vectors be A and consider the convex hull of A. As matrices, we see that $conv(A)$ is the set of matrices in $\mathbb{R}^{m \times n}$ with the same row and column sums as $\mathcal{B}(\mathcal{R})$, with all entries constrained between 0 and 1.

From this set of matrices, we find the matrix M_{max} that maximizes the entropy function

$$H(M) = \sum_{i,j} \left(M_{ij} \ln \frac{1}{M_{ij}} + (1 - M_{ij}) \ln \frac{1}{1 - M_{ij}} \right)$$

on $conv(A)$. The function H is strictly concave and $conv(A)$ is a convex set which guarantees a unique solution to the maximization. The matrix M_{max} is then used as a matrix of probabilities where $P(B^*_{ij} = 1)$ is equal to $(M_{max})_{ij}$.

Once we have the matrix of probabilities $P_{ij} = P(B^*_{ij} = 1)$ we note that, since $G^* = B^* B^{*\top}$,

$$G^*_{ij} = B^*_{i1} B^*_{j1} + B^*_{i2} B^*_{j2} + \cdots + B^*_{in} B^*_{jn}.$$

Furthermore, since the probabilities are independent,

$$P(B^*_{ik} B^*_{jk} = 1) = P(B^*_{ik} = 1) P(B^*_{jk} = 1) = P_{ik} P_{jk}.$$

It follows that G^*_{ij} is Poisson binomial with parameters $p_1 = P_{i1} P_{j1}, \ldots, p_n = P_{in} P_{jn}$.

Computing the exact cumulative distribution function for a Poisson binomial distribution is difficult. However, the Poisson binomial distribution is well-approximated by a refined normal distribution [17], which we use in the stochastic degree sequence model.

Fixed Degree Sequence Model (FDSM)

The fixed degree sequence model constrains both the row and column sums in B^* to be equal to their values in B (i.e. both are fixed) [19]. The probability distribution that

[1] Although [16] suggested using the Poisson binomial distribution in this case, their equation (6) for computing the probability that two given primary nodes are connected to a given secondary node can sometimes yield numbers great than 1. We provide a correct proof here.

[2] Although this approach is simple, fast, and recommended by multiple authors, it can yield values greater than 1. In practice, these values are often truncated to 1 for use as probabilities.

describes $(B^*B^{*\top})_{ij}$ for all bipartite graphs $B^* \in \mathcal{B}(\mathcal{R})$ is unknown, and thus an approximate distribution is constructed via simulation:

1. Construct a bipartite graph B^* that represents a random draw from $\mathcal{B}(\mathcal{R})$.
2. Project B^* (i.e. compute $B^*B^{*\top}$) to obtain a random weighted bipartite projection G^*.
3. Repeat steps 1 and 2 N times to sample the space of possible G^*_{ij}.

The matrix multiplication required in step 2 is computationally expensive but straightforward [20]. However, the random sampling of a B^* from $\mathcal{B}(\mathcal{R})$ in step 1 is more challenging. Several methods have been suggested, including Markov Swap methods [21] and Sequential Importance Sampling [22], but in the fixed degree sequence model we use the curveball algorithm [23], which is one of the fastest algorithms that has been proven to randomly sample [24, see also [25]].

Applying `backbone` to the US Senate

We illustrate the use of the R `backbone` package to extract the backbone of a network of bill co-sponsorship relations among Senators in the 114th session of the United States Senate. This context provides insight into how the `backbone` package works because both prior research [26] and media accounts [27] of the current US political climate provide us with *a priori* expectations about what structure a properly extracted backbone should have. Specifically, given conditions of partisanship and polarization, we should expect to see positive relationships form primarily between those in the same political party. In visualizations of the extracted backbones, we depict Republican senators by red vertices, Democratic senators by blue vertices, and Independent senators by green vertices. Although we discuss signed backbones in the text, for visual clarity only provide figures for binary backbones which contain positive edges. Positive relations of collaboration between two Republicans are red, between two Democrats are blue, and for all other pairs are gray.

Data

The data consists of 100 senators and the 3589 bills that they have sponsored in the 114th session of Congress [28][3]. This data takes the form of a bipartite graph B, where the two independent sets of vertices are the senators (agents) and the bills (artifacts). Each edge connects one senator to one bill. Specifically, $B_{ij} = 1$ if senator i sponsored bill j, and otherwise is 0. Below we examine the data set. Notice that the row names correspond to each senator (including their party affiliation and the state they represent) and the column names refer to the bill number.

```
set.seed(19)
library(backbone)
senate <- read.csv("S114.csv", row.names = 1, header = TRUE)
senate <- as.matrix(senate)
dim(senate)
```

```
## [1]  100 3589
```

[3] These data include all senators who served at *any* time during the 114th session. We use "sponsor" to generically refer to all legislators who have signed onto a bill.

```r
senate[1:5, 1:5]
```

```
##                        sj9 sj8 sj7 sj6 sj5
## Alexander, L. (TN-R)   0   1   0   1   0
## Boxer, B. (CA-D)       0   0   0   0   1
## Cantwell, M. (WA-D)    0   0   0   0   1
## Carper, T. (DE-D)      0   0   0   0   1
## Cochran, T. (MS-R)     0   1   0   1   0
```

A weighted graph G can be constructed from B via bipartite projection, where $G = BB^{\top}$ and G_{ij} contains the number of bills that both senator i and senator j sponsored. Notice the graph is now 100 rows by 100 columns.

```r
G <- senate%*%t(senate)
dim(G)
```

```
## [1] 100 100
```

```r
G[1:5, 1:2]
```

```
##                        Alexander, L. (TN-R) Boxer, B. (CA-D)
## Alexander, L. (TN-R)                    141               10
## Boxer, B. (CA-D)                         10              303
## Cantwell, M. (WA-D)                      15               82
## Carper, T. (DE-D)                        12               55
## Cochran, T. (MS-R)                       40               25
```

The projected graph G now indicates that Senator Lamar Alexander sponsored a total of 141 bills in the 114th session. Among these 141 bills, 10 were co-sponsored by Senator Barbara Boxer, and 15 were co-sponsored by Senator Maria Cantwell.

We can use the values of graph G to observe differences between those with similar or dissimilar ideology. Below, we compare the number of bills co-sponsored by two individuals with similar political ideology, Senators Cory Booker and Elizabeth Warren, versus those with dissimilar ideology, Senators Ted Cruz and Bernie Sanders. The results are consistent with the expectation that legislators sharing a similar ideology engage in more co-sponsorships.

```r
G["Booker, C. (NJ-D)", "Warren, E. (MA-D)"]
```

```
## [1] 98
```

```r
G["Cruz, T. (TX-R)", "Sanders, B. (VT-I)"]
```

```
## [1] 5
```

The differences in the number of bills co-sponsored prompts an important underlying question: how many bills do two senators have to co-sponsor before we would be justified in concluding they are political collaborators? Similarly, how few bills do they have to co-sponsor before we would be justified in concluding they are political opponents? These questions are what the `backbone` package seeks to answer.

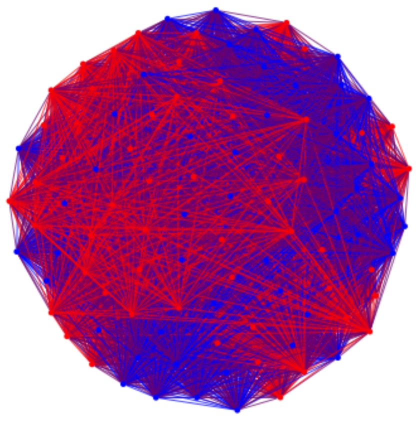

Fig 1. The positive backbone of the US Senate co-sponsorship network with edges retained between two senators if they sponsored at least 1 bill together.

Extracting a universal threshold backbone: universal( )

The simplest approach to backbone extraction applies a single threshold T to all edges. A threshold value of 0 indicates that as long as a pair of vertices are affiliated with at least one of the same artifacts (i.e. they have a nonzero edge weight), their relationship should be counted. However, this can lead to extremely dense and uninformative networks, as we will show with our example data. In any case, if using a single threshold value T is desired, this can be done in the `backbone` package by using the `universal()` function. This function allows the user to extract an unweighted backbone by selecting a single threshold T, or extract a signed backbone by selecting upper and lower thresholds T^+ and T^-.

Using the `senate` data set, we use the `universal()` function to compute a backbone with a single threshold of 0. Thus if two senators have cosponsored one or more bills, there will be an edge between them. Notice that our backbone graph is represented by a square adjacency matrix with 0-1 entries.

```
universal_bb1 <- universal(senate, upper = 0, bipartite = TRUE)
universal_bb1$backbone[1:5, 1:2]
```

```
##                      Alexander, L. (TN-R) Boxer, B. (CA-D)
## Alexander, L. (TN-R)                    0                1
## Boxer, B. (CA-D)                        1                0
## Cantwell, M. (WA-D)                     1                1
## Carper, T. (DE-D)                       1                1
## Cochran, T. (MS-R)                      1                1
```

We plot our upper threshold of 0 using the `igraph` package [29]. Note that the resulting graph in fig. 1 is extremely dense as only 1 pair of senators out of the total 4950 unique pairs have not sponsored at least one bill together. The universal threshold backbone is uninformative about the underlying structure of the network.

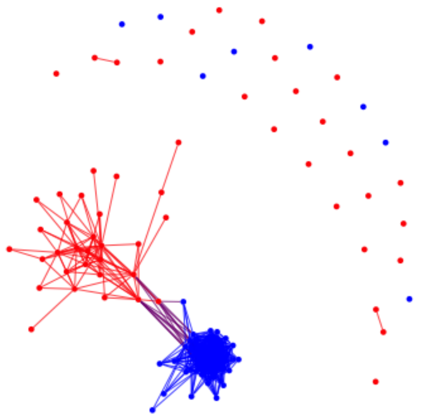

Fig 2. The positive backbone of the US Senate co-sponsorship network with edges retained between two senators if they sponsored more bills together than one standard deviation above the mean.

To create a signed backbone, we can apply both an upper and lower threshold value. The following code will return a backbone where the positive edges indicate two senators cosponsored more than 1 standard deviation above the mean number of co-sponsored bills and negative edges indicate two senators cosponsored less than 1 standard deviation below the mean number of co-sponsored bills. The graph of the positive edges of this backbone can be seen in fig. 2.

```
universal_bb2 <- universal(senate, upper = function(x) mean(x)+sd(x),
    + lower = function(x) mean(x)-sd(x), bipartite = TRUE)
```

Although the resulting graph in fig. 2 is much less dense than when using an upper threshold of 0, it still does not necessarily reveal the underlying structure of the network among legislators. In this case, “the application of a threshold to the global weight distribution...belittles nodes with a small [degree],” resulting in a backbone that preserves edges only among legislators who co-sponsor many bills, and treating legislators who co-sponsor few bills as isolates [30, p. 6484]. To obtain meaningfully sparse graphs that do not ignore the multi-scalar character of node degrees we must allow the threshold to vary for different edges. To improve our backbone results, we move to methods of bipartite projections which determine a distinct threshold value for each pair of vertices.

Extracting a null model backbone: backbone.extract( )

Instead of using a universal threshold to determine a backbone, the `backbone` package permits using a model that is based on a statistical test, such as the hypergeometric model, stochastic degree sequence model, or fixed degree sequence model. To use these methods in `backbone`, one first calls to the method (`hyperg()`, `sdsm()`, or `fdsm()`) which finds the probability of observing an edge with the observed weight in a corresponding null model, returning an object of class ‘backbone.’ This object is then

supplied to `backbone.extract()`, which performs the hypothesis test for a given significance value and returns a backbone graph. The user can input bipartite graph objects of class 'matrix', 'sparseMatrix', 'Matrix', 'igraph', 'network', and 'edgelist' (a matrix of two columns), and can choose the type of backbone returned by specifying the desired class in `backbone.extract()`.

The `backbone.extract()` function allows the user to input the backbone class object and obtain either a signed or positive backbone. The `backbone.extract()` function has five arguments: `matrix`, `signed`, `alpha`, `class`, `narrative`, and `fwer`. The `matrix` argument takes a backbone object generated by `hyperg()`, `sdsm()`, or `fdsm()` and returns a backbone graph of class = `class` using a two-tailed significance test with significance value $\alpha =$ `alpha`. If the `signed` parameter is set to `TRUE` then a signed backbone is returned, if it is set to `FALSE` then a positive backbone is returned. If the `narrative` parameter is set to `TRUE` then suggested narrative text for a manuscript, including possible citations, is displayed.

Extracting the backbone of a bipartite projection involves conducting an independent statistical test on $\ell = m(m-1)/2$ edges in the projection, where m is the number of vertices in the bipartite projection. Because each of these tests is independent, this can inflate the familywise error rate beyond the desired `alpha`. The `fwer` parameter offers two ways to correct for this: the classical Bonferroni correction is applied when `fwer = 'bonferroni'`, and the more powerful Holm-Bonferroni correction is applied when `fwer = 'holm'` [31].

Hypergeometric backbone: hyperg( )

To apply the hypergeometric distribution to a bipartite graph, one uses the `hyperg()` function. The `hyperg()` function returns a backbone class object that contains: a `positive` matrix with (i, j) entry equal to the probability that G^*_{ij} is equal to or above the corresponding entry in G, and a `negative` matrix with (i, j) entry equal to the probability that G^*_{ij} is equal to or below the corresponding entry in G, and a `summary` data frame with includes the name of the model, number of rows and columns in the adjacency matrix of the input graph, skew of the row and columns, and running time of the model.

```
hyperg_probs <- hyperg(senate)
```

```
## Finding the distribution using hypergeometric distribution
```

```
hyperg_bb <- backbone.extract(hyperg_probs, alpha = .01)
```

We can now examine how this method has changed the appearance of our network, focusing only on the positive edges of the signed backbone in fig. 3. We can see that the hypergeometric function has reduced the density of our network and that we begin to see some of the two party structure that is inherent in the United States Senate.

Specifically, for our example, the hypergeometric function will fix the number of bills that each senator sponsors, while allowing each bill to be sponsored by a varying number of senators. The function will compute the probability of each senator sponsoring at least (or at most) the observed number of bills when the bills which they sponsor were chosen randomly.

The stochastic degree sequence model: sdsm( )

The `sdsm()` function returns a backbone object containing the same objects as `hyperg()`: matrices `positive` and `negative` containing probabilities under the stochastic degree sequence model, as well as a `summary` data frame.

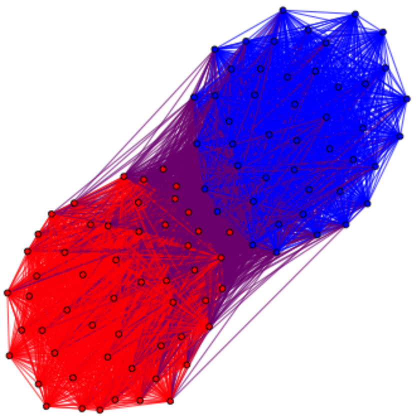

Fig 3. The positive backbone of the US Senate co-sponsorship network under the hypergeometric model.

In the context of the senate co-sponsorship matrix, the stochastic degree sequence model will compare our observed values to a distribution where each senator sponsors roughly the same number of bills, and each bill is sponsored by roughly the same number of people.

Here we use the `polytope` model to compute the probabilities for the Poisson binomial distribution.[4]

```
sdsm <- sdsm(senate, model = "polytope")
```

```
## Finding the distribution using SDSM with polytope model.
```

```
sdsm_bb <- backbone.extract(sdsm, narrative = TRUE, alpha = .01)
```

```
## Suggested manuscript text and citations:

From a bipartite graph containing 100 agents and 3589 artifacts, we
obtained the weighted bipartite projection, then extracted its signed
backbone using the backbone package (Domagalski, Neal, & Sagan, 2020).
Edges were retained in the backbone if their weights were statistically
significant (alpha = 0.01) by comparison to a null Stochastic Degree
Sequence Model (Neal, 2014).

Domagalski, R., Neal, Z. P., and Sagan, B. (2020).  backbone:  An R
Package for Backbone Extraction of Weighted Graphs.  arXiv:1912.12779
[cs.SI]
```

[4]Occasionally `backbone` will report that the polytope results are not optimal. This suboptimality is related to computational precision, however because the polytope results are used to compute an approximate Poisson binomial distribution with a large number of parameters, such suboptimal precision likely has little practical impact.

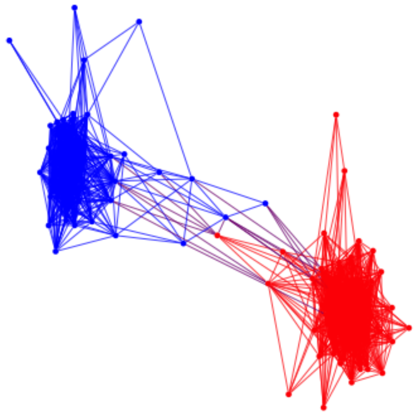

Fig 4. The positive backbone of the US Senate co-sponsorship network under the stochastic degree sequence model.

```
Neal, Z. P. (2014).  The backbone of bipartite projections:  Inferring
relationships from co-authorship, co-sponsorship, co-attendance and
other co-behaviors.  Social Networks, 39, 84-97.
https://doi.org/10.1016/j.socnet.2014.06.001
```

We are able to see more of the partisan structure that is suggested to be present in the US Senate in fig. 4, and this visualization provides more information than the extremely dense graph found using a universal threshold.

The fixed degree sequence model: fdsm( )

The fixed degree sequence model first constructs a random bipartite graph B^* that preserves both degree sequences using the curveball algorithm [12]. This bipartite graph B^* is then projected to obtain a random weighted bipartite projection $G^* = B^* B^{*\top}$. These two steps are repeated a number of times to sample the space of possible G^*_{ij}. At each iteration, we compare G_{ij} to the value of G^*_{ij} and keep a record of how often it was above, below, or equal to the generated value. The `fdsm()` function returns a backbone object containing a matrix object `positive` of the proportion of times G^*_{ij} is equal to or above the corresponding entry in G, and a matrix object `negative` containing the proportion of times G^*_{ij} is equal to or below the corresponding entry in G, and a `summary` data frame as in `hyperg()` and `sdsm()`.

The function can also save each value of G^*_{ij} for a given i, j. This is useful for visualizing an example of the empirical null edge weight distribution generated by the model. The values i, j correspond to the row and column indices of a cell in the projected matrix and can be input as either numeric values or a string containing the row names. These values are returned in the list `dyad_values`.

Using the fixed degree sequence model on the senate data set will allow us to compare our observed values to a distribution where each senator sponsors the exact same number of bills and each bill is sponsored by the exact same number of people. We can find the backbone using the fixed degree sequence model as follows:

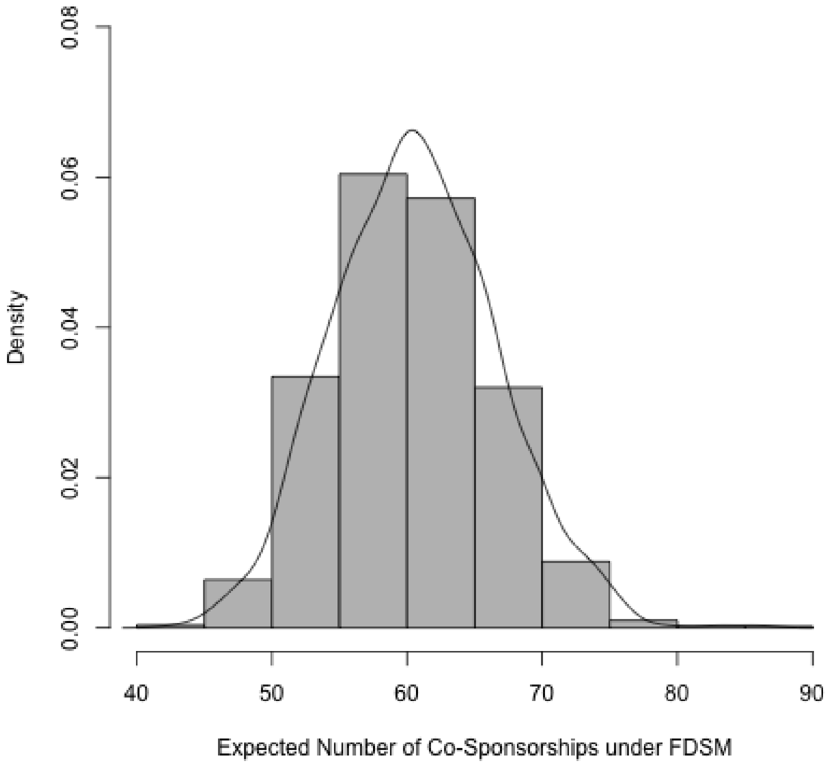

Fig 5. A histogram of the expected co-sponsorships between Senators Cory Booker and Elizabeth Warren under the fixed degree sequence model (1000 samples). A positive edge between Booker and Warren would be preserved in the FDSM backbone because their actual number of co-sponsorships (98) is statistically significantly larger.

```
fdsm <- fdsm(senate, trials = 1000, dyad = c("Booker, C. (NJ-D)",
     + "Warren, E. (MA-D)"))
```

```
## Approximating the distribution using Curveball FDSM
## Estimated time to complete is 127 secs
```

```
hist(fdsm$dyad_values, freq = FALSE, xlab = "Number of Co-Sponsorships")
lines(density(fdsm$dyad_values))
```

The `dyad_values` output is a list of the G^*_{ij} values for each of the 1000 trials, where i = "Booker, C. (NJ-D)" and j = "Warren, E. (MA-D)". These values correspond to the number of bills Senators Booker and Warren would be expected to co-sponsor when we create a random bipartite graph with the curveball algorithm where: (a) the number of bills sponsored by Senator Booker, by Senator Warren, and all other Senators was fixed, and (b) the number of senators sponsoring each bill was fixed. We can compare their actual number of co-sponsorships, 98, to what is generated under our null model. We can view a histogram of the expected co-sponsorships generated in each of the 1000 trials in fig. 5.

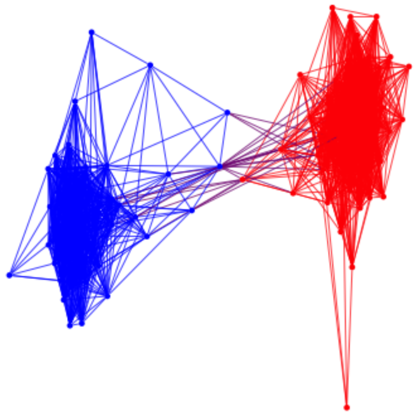

Fig 6. The positive backbone of the US Senate co-sponsorship network under the fixed degree sequence model.

To extract the backbone, we supply the `backbone.extract()` function with the proportion matrices `positive` and `negative`.

```
fdsm_bb <- backbone.extract(fdsm, alpha = 0.01, signed = TRUE)
```

Using the fixed degree sequence model allows us to see more of th[illegible]artisan structure we assume to be present in the United States Senate in fig. [illegible]

Discussion

We have presented four methods – universal threshold, HM, SDSM, and FDSM – for identifying significant links in a weighted bipartite projection, and thus for extracting its binary or signed backbone. We have also introduced and demonstrated the R `backbone` package, which implements these methods in a common framework facilitating their use. Together, the methods and package offer ways for researchers to reduce the structural artifacts (e.g., excessive density and clustering) and complexity of bipartite projections, making them easier to analyze and visualize. This is likely to be most useful in cases where the researcher is interested in an unobserved unipartite network that is not simply a bipartite projection of something (e.g., a social network), but that can be inferred (even with some error) from observed bipartite data (e.g., co-behaviors).

There are two important cases where `backbone` is not appropriate and should *not* be used. First, if the research question can be answered by directly analyzing the original bipartite graph, or the central message can be communicated by visualizing the original bipartite graph, then it is not necessary and indeed may be harmful to examine a bipartite projection or its backbone. In these cases, researchers should instead use tools designed for the analysis of bipartite networks, including for example bipartite extensions of ERGM [32] and SIENA [33]. Second, it is possible to obtain a weighted (via projection) or unweighted (via `backbone`) square symmetric matrix from *any* bipartite data, however this does not imply that the matrix can be meaningfully

analyzed as a network. Treating a square symmetric matrix obtained via projection or backbone extraction as a network requires an *explicit conceptual* rationale for why the sharing of artifacts by a pair of agents provides information about those agents' relationship with each other, and a description of what kind of relationship this is. In some cases, the rationale may be simple and straightforward: social events and other venues offer opportunities for people to meet and interact, so observing two people attending many of the same events, may indirectly provide information about the possibility of a social relationship between them [1]. In other cases – for example, psychological networks in which clinical symptoms (the 'agents') are linked because they co-occur in patients (the 'artifacts') [34] – the rationale is far from obvious [35].

Even when backbones of bipartite projections are used in appropriate ways, there remain a number of open questions that should guide future research in this area and future development of the `backbone` package. First, although the `backbone` package implements four methods for extracting the backbone of bipartite projections, we know little about how the backbones generated by these methods differ from one another. Second, to the extent that these methods are intended to provide insight into an unobserved but underlying unipartite network, we know little about which backbone extraction methods do so most accurately. As a result, although there are known problems associated with the use of a universal threshold, it is difficult to know how to choose among other backbone extraction methods. However, the availability of the `backbone` package makes it possible to begin answering these questions, while also providing a platform for implementing methods and exploring similar questions in the more general case of extracting the backbone of non-projection weighted graphs.

Acknowledgments

This work was supported by the National Science Foundation (#1851625 & #2016320).

Author contributions

RD and ZN wrote and tested the `backbone` package, BS conceptualized the polytope-based SDSM probabilities, RD drafted the initial manuscript, all authors revised the manuscript.

References

1. Breiger RL. The duality of persons and groups. Social forces. 1974;53(2):181–190.

2. Neal ZP. A sign of the times? Weak and strong polarization in the US Congress, 1973–2016. Social Networks. 2020;60:103–112.

3. Heemskerk EM, Fennema M, Carroll WK. The global corporate elite after the financial crisis: evidence from the transnational network of interlocking directorates. Global Networks. 2016;16(1):68–88.

4. Newman ME. Scientific collaboration networks. I. Network construction and fundamental results. Physical review E. 2001;64(1):016131.

5. Leydesdorff L, Vaughan L. Co-occurrence matrices and their applications in information science: Extending ACA to the Web environment. Journal of the American Society for Information Science and technology. 2006;57(12):1616–1628.

6. Zhang B, Horvath S. A general framework for weighted gene co-expression network analysis. Statistical applications in genetics and molecular biology. 2005;4(1).

7. Latapy M, Magnien C, Del Vecchio N. Basic notions for the analysis of large two-mode networks. Social networks. 2008;30(1):31–48.

8. Watts DJ. Six Degrees: The Science of a Connected Age. W. W. Norton; 2008.

9. Neal Z. The backbone of bipartite projections: Inferring relationships from co-authorship, co-sponsorship, co-attendance and other co-behaviors. Social Networks. 2014;39:84–97. doi:10.1016/j.socnet.2014.06.001.

10. R Core Team. R: A Language and Environment for Statistical Computing; 2018. Available from: https://www.R-project.org.

11. Domagalski R, Neal Z, Sagan B. backbone: Extracts the Backbone from Weighted Graphs; 2020. Available from: https://CRAN.R-project.org/package=backbone.

12. Strona G, Ulrich W, Gotelli NJ. Bi-dimensional null model analysis of presence-absence binary matrices. Ecology. 2018;99(1):103–115. doi:10.1002/ecy.2043.

13. Gotelli NJ. Null model analysis of species co-occurrence patterns. Ecology. 2000;81(9):2606–2621.

14. Tumminello M, Miccichè S, Lillo F, Piilo J, Mantegna RN. Statistically Validated Networks in Bipartite Complex Systems. PLOS ONE. 2011;6(3):e17994. doi:10.1371/journal.pone.0017994.

15. Neal Z. Identifying statistically significant edges in one-mode projections. Social Network Analysis and Mining. 2013;3(4):915–924. doi:10.1007/s13278-013-0107-y.

16. Liebig J, Rao A. Fast extraction of the backbone of projected bipartite networks to aid community detection. Europhysics Letters. 2016;113(2):28003. doi:10.1209/0295-5075/113/28003.

17. Hong Y. On computing the distribution function for the Poisson binomial distribution. Computational Statistics & Data Analysis. 2013;59:41–51. doi:10.1016/j.csda.2012.10.006.

18. Barvinok A. Combinatorics and complexity of partition functions. vol. 9. Springer; 2016.

19. Zweig KA, Kaufmann M. A systematic approach to the one-mode projection of bipartite graphs. Social Network Analysis and Mining. 2011;1(3):187–218. doi:10.1007/s13278-011-0021-0.

20. Le Gall F. Powers of tensors and fast matrix multiplication. In: Proceedings of the 39th international symposium on symbolic and algebraic computation; 2014. p. 296–303.

21. Gionis A, Mannila H, Mielikäinen T, Tsaparas P. Assessing data mining results via swap randomization. ACM Transactions on Knowledge Discovery from Data (TKDD). 2007;1(3):14–es.

22. Chen Y, Diaconis P, Holmes SP, Liu JS. Sequential Monte Carlo methods for statistical analysis of tables. Journal of the American Statistical Association. 2005;100(469):109–120.

23. Strona G, Nappo D, Boccacci F, Fattorini S, San-Miguel-Ayanz J. A fast and unbiased procedure to randomize ecological binary matrices with fixed row and column totals. Nature Communications. 2014;5:4114. doi:10.1038/ncomms5114.

24. Carstens CJ. Proof of uniform sampling of binary matrices with fixed row sums and column sums for the fast Curveball algorithm. Physical Review E. 2015;91(4). doi:10.1103/PhysRevE.91.042812.

25. Wang G, et al. A fast MCMC algorithm for the uniform sampling of binary matrices with fixed margins. Electronic Journal of Statistics. 2020;14(1):1690–1706.

26. Layman GC, Carsey TM, Horowitz JM. Party polarization in American politics: Characteristics, causes, and consequences. Annu Rev Polit Sci. 2006;9:83–110.

27. Drutman L. American politics has reached peak polarization; 2016. Available from: https://www.vox.com/polyarchy/2016/3/24/11298808/american-politics-peak-polarization.

28. USGPO. govinfo – Bulk Data - Bill Status. United States Government Publishing Office (GPO). 2020;.

29. Csardi G, Nepusz T. The `igraph` software package for complex network research; 2006. Available from: http://igraph.org.

30. Serrano MÁ, Boguná M, Vespignani A. Extracting the multiscale backbone of complex weighted networks. Proceedings of the national academy of sciences. 2009;106(16):6483–6488.

31. Holm S. A simple sequentially rejective multiple test procedure. Scandinavian journal of statistics. 1979; p. 65–70.

32. Wang P, Pattison P, Robins G. Exponential random graph model specifications for bipartite networks—A dependence hierarchy. Social networks. 2013;35(2):211–222.

33. Koskinen J, Edling C. Modelling the evolution of a bipartite network—Peer referral in interlocking directorates. Social Networks. 2012;34(3):309–322.

34. Borsboom D, Cramer AO. Network analysis: an integrative approach to the structure of psychopathology. Annual review of clinical psychology. 2013;9:91–121.

35. Bringmann LF, Eronen MI. Don't blame the model: Reconsidering the network approach to psychopathology. Psychological Review. 2018;125(4):606.
