# Supplementary material for: Backbone: An R package for extracting the backbone of bipartite projections
Source: PLoS One. 2021 Jan 6;16(1):e0244363. doi: 10.1371/journal.pone.0244363 (PMC7787471; doi:10.1371/journal.pone.0244363)
Supplement: Attachment — Submitted filename: Letter_.pdf [file pone.0244363.s003.pdf]

Summary of revisions to:
"backbone: An R package for extracting the backbone of bipartite projections"
(Manuscript PONE-D-20-23636)

Dear Dr. Rozenblat,

Thank you for the very helpful comments from these three reviewers, and for the opportunity to revise and resubmit. Below we provide detailed responses to each of the reviewers' comments.

1 Model differentiation and selection (all reviewers)

Reviewer 1: The most important part of the paper, though, is given in the last paragraph (line 424-436). None of the implemented backbone extraction methods can be considered superior and there are, so far, no theoretical or technical guidelines for which method to choose in which context.
Reviewer 2: We...encourage the authors to go a little further in analysing the differences between the different methods proposed and the different backbones obtained.
Reviewer 3: It would be helpful to further compare how each method differs from each other from a computational perspective. The only part of the paper where this appears is when the authors mention the computational cost of the FSDM (page 6). This point should itself be further documented, e.g. by giving some idea of how long it would take to apply that method on a much larger network, since much larger networks are very common in complex network analysis. It might also be useful to show that the other methods documented in the paper remain computationally inexpensive on much higher network sizes and under varying densities, unless one considers this to be trivial enough to be left out.
All three reviewers requested greater detail on how the backbone models implemented in the `backbone` package differ, and how a user should choose a particular model. In this revision, we have addressed this helpful suggestion in two ways:

- For each backbone extracted in our example, we now present both the running time and modularity, which together provide indications of the model's practicality and accuracy.
- We have added a new section titled "Backbone model comparison and selection" in which we compare the example backbones, and provide specific guidance on backbone model selection.

2 Reviewer 1

2.1 The paper is well organized and explains the package structure well.

Thank you for your positive comments; we are glad that you enjoyed the paper.

2.2 Line 143: paramenters-¿parameters

Corrected

3 Reviewer 2

3.1 We salute the quality of the writing and the clarity of the demonstrations.

Thank you for your positive comments. We are happy to hear that you found the demonstrations clear and easy to follow.

3.2 Line 16: "when transforming a bipartite graph into a unipartite graph via projection, information about the 'events' responsible for edges between vertices is lost." This is not entirely true since one can partly keep this information by weighting the links accordingly: see https://toreopsahl.com/tnet/two-mode-networks/projection/

We have clarified this by noting that "in particular, one no longer knows *which* event(s) gave rise to a given edge." We now also include as a footnote: "There exist some edge weighting schemes that aim to encode some information about the events into the edge weights of the unipartite projection (Newman, 2001)"

3.3 Line 21-24: "observing two people attending many of the same events (i.e. a large edge weight) may still be uninteresting if they each attended many large events, but observing two people attend just a few of the same events (i.e. a small edge weight) may be interesting if they each attended only a few small events."Weighting methods such as the ones mentioned in the link above offer to deal with such an issue. To what extend would you say that backbones extraction offer a better way to cope with this?

The weighting methods discussed by Newman (2001) provide ways to indirectly encode some information about 'events' in the edge weights of a weighted bipartite projection, but they still do not provide a way to interpret the magnitude of those weights. In contrast, the backbone methods we discuss here offer a formal statistical test of the significance of an edge weight's magnitude compared to several different null models.

3.4 Line 61-65: "Bipartite projections are of interest in social network analysis because they allow us to construct a network from artifact affiliations, which are often easier to obtain than taking a survey of the network members. If the number of members of the network is large, getting complete and reliable information regarding relationships between members can involve long and repetitive survey techniques which can lead to survey fatigue and costly field work." Note that it is true for social network analysis in sociology... but in history, for instance this possibility (taking a survey of the network members) is not even possible.

This is an excellent point. We now note: "Moreover, in historical contexts a survey of long-deceased network members is simply not possible."

3.5 Line 70-71: "Because bipartite projections are weighted, and because what counts as a 'large' or 'small' weight can differ for each edge, it can be useful to reduce this information." I understand that you refer to the idea developed in the introduction regarding the difference between large and small events in the case of events co-attendance, but maybe the wording could be more explicit here, perhaps with an example.

We have expanded our discussion of the challenges with bipartite projections in the introduction, and now provide multiple examples for the case of large vs. small events, and the case of many vs. few events.

3.6 Line 136-137: "That is, for any given B*, each row and column sum may be higher or lower than its observed degree in B, but on average they are equal." Again, I'm not sure that the formulation is clear enough here, it seems to me that this sentence can be understood in different ways.

We have clarified this sentence as: "That is, for any given B^*, each row and column sum may be higher or lower than what is observed in B, but if one takes the average over all the possible B^* of the sum of a given row or column then one obtains the corresponding sum in B."

3.7 Line 193-195: "In visualizations of the extracted backbones, we depict Republican senators by red vertices, Democratic senators by blue vertices, and Independent senators by green vertices." Actually, we can't see any green on the figures. In the Replication Code, we see that democrats and independents are plotted in the same color because of this line (l. 16): `V(iB)$party[V(iB)$party == "I"] <- "D"`

This was an oversight from an earlier version of the code and visualizations. We have corrected the text noting "...and both Democratic and Independent senators by blue vertices" and have added this explanatory footnote: "Sen. Bernie Sanders of Vermont and Sen. Angus King of Maine are both Independents, however they hold left-leaning ideologies and caucus with the Democrats.'

3.8 Line 196: "Although we discuss signed backbones in the text, for visual clarity only provide figures for binary backbones which contain positive edges." Missing "we" before "provide".

Corrected

3.9 Line 196-198: "Positive relations of collaboration between two Republicans are red, between two Democrats are blue, and for all other pairs are gray." The other pairs look rather purple to me.

We have corrected the text to note that bipartisan collaborations (i.e. collaborations between a republican and a democrat or independent) are purple.

3.10 Line 235-239: "The differences in the number of bills co-sponsored prompts an important underlying question: how many bills do two senators have to co-sponsor before we would be justified in concluding they are political collaborators? Similarly, how few bills do they have to co-sponsor before we would be justified in concluding they are political opponents? These questions are what the backbone package seeks to answer." In addition to plotting the backbones resulting from the different methods, it would be interesting to find a way to compare the answers that each extraction method can possibly give to these questions in the case of the 114th session of the US Senate. This could contribute to respond to the more general question addressed in the discussion part of the paper: how can we know which extraction method is the more relevant for the research question we ask?

Each backbone extraction model (hypergeometric, SDSM, and FDSM) yield a potentially unique threshold for each edge. In figure 5 we illustrate the numerically estimated probability distribution for one edge (between Booker and Warren) from one model (FDSM). It would go beyond the scope of this paper to compare the probability distributions for every edge from every model (there would be nearly 15,000 such distributions). However, we do now quantitatively compare the backbones yields by each model and comment on how users should choose among the available models.

3.11 Line 356-357: "We are able to see more of the partisan structure that is suggested to be present in the US Senate in fig. 4." Line 392-393: "Using the fixed degree sequence model allows us to see more of the partisan structure we assume to be present in the United States Senate in fig. 6." The argument given to comment on Figures 5 and 6 is identical, which is rather disappointing for interpreting the difference between the results obtained using the two distinct methods. To go further, could you give more details on the difference between this backbone (displayed on fig. 6) and the previous one (displayed on fig. 4), and try answering the following question: which one performs the most in displaying the expected partisan structure? And why is it so?

We have added a new section in which we explicitly compare backbone models and the backbone networks they generate. In this section, we explain when and why backbone networks from different models will be different, and how users should choose one model over another.

3.12 Line 430-431: "Second, to the extent that these methods are intended to provide insight into an unobserved but underlying unipartite network, we know little about which backbone extraction methods do so most accurately." You could start giving us some clues about this using the example of bill co-sponsorships.

In this revision, we use the modularity statistic as an indicator of the potential validity of these backbone networks.

3.13 Reference 18: Error in the volume number, it is volume 30 and not volume 9.

Corrected

4 Reviewer 3

4.1 The paper stands very well as it is – it is clear, straightforward, and rich in details on the software implementation of the method, which will help others to build on it.

Thank you. It was our goal to offer this software and paper as a starting point for others.

4.2 The paper states to implement the "most common" (page 2) bipartite backbone extraction methods, but the commonality of the methods covered by the package is undocumented in the paper. Perhaps the paper should more readily redirect the reader to ref. 9 (Neal 2014), in order to clearly indicate that the commonality of the methods covered by the package is demonstrated in this paper.

We have revised this sentence by replacing "most common" with "these": "Multiple methods of backbone extraction exist for bipartite projections (Neal, 2014), but until now there has not been a single software package that implements **these** methods."

4.3 The paper does not document parameter tuning, i.e. how much of the total weights, nodes and edges are preserved in the backbone when parameter alpha is lowered from e.g. 0.05 to 0.001. A good example of that is found in Serrano et al. 2009 (ref. 30 in the present paper). The same paper also documents how that parameter influences the network topology of the resulting backbone. Doing so would enhance the example part of the paper, which is weak in comparison to the other (very good) papers published by the main author on the topic of Congressional political polarization measured through bill co-sponsorship (in Sci. Rep. 2020 and in Soc. Net. 2020).

Thank you for this helpful suggestion. We are currently pursuing this question in another manuscript. For this reason, and because the manuscript is already 17 single-spaced pages, we have chosen to keep this manuscript focused on demonstrating the `backbone` package itself, rather than formally examining the statsitical properties of the models implemented in the package.
